# Evaluation and Strategic Response of Sustainable Livelihood Level of Farmers in Ecological Resettlement Area of the Upper Yellow River—A Case Study of Liujiaxia Reservoir Area, Gansu Province

**DOI:** 10.3390/ijerph192416718

**Published:** 2022-12-13

**Authors:** Zongxiang Wang, Wei Li, Jianwu Qi

**Affiliations:** 1 College of Geography and Environmental Science, Northwest Normal University, Lanzhou 730070, China; 2 Institute of Urban Planning and Tourism Landscape Design, Northwest Normal University, Lanzhou 730070, China; 3 Department of Tourism Management, South China University of Technology, Guangzhou 510006, China

**Keywords:** ecological resettlement area, sustainable livelihoods, evaluation system, coupling coordination degree, space differentiation

## Abstract

The level of sustainable livelihoods, as a yardstick for measuring the social development of migrants, is of great importance to the sustainable development of the region. Based on the analysis of the policy logic of ecological protection and high-quality development in the Yellow River basin, this paper constructs a “ternary” system model and evaluation index system for sustainable livelihoods of farm households in the ecological resettlement areas of the upper Yellow River, and proposes that the harmonious relationship between the three basic dimensions of economy, society and environment is the key to evaluate the sustainable livelihood level of farm households in ecological resettlement areas. Based on the comprehensive evaluation index to assess the comprehensive development level of ecological resettlement areas, we introduced the coupling coordination degree and constructed the coordinated development degree model of “economic-social-environmental” system to characterize the sustainable livelihood level. Through the data of 1116 questionnaires and in-depth interviews in the ecological migrant resettlement area of Liujiaxia reservoir in the upper reaches of the Yellow River basin, the sustainable livelihood status and spatial distribution differences of farm households in 14 townships in the region were evaluated, and the validity of the indicator system was empirically tested. Finally, sustainable livelihood strategies for farm households in the ecological resettlement areas of the upper Yellow River are proposed for the economic, social and environmental dimensions, and the indicator system is further revised. The evaluation system can not only advance the research paradigm of sustainable livelihood assessment for farmers in ecological migrant resettlement areas but can also be widely guided and applied to the sustainable development of ecological migrant practices in China.

## 1. Introduction

The development of human society is always accompanied by migration and change. At present, how to alleviate the acute contradiction between the survival and development needs of human beings and the production and recovery capacity of the environment has become an urgent global problem for all countries to solve [1]. For half a century, as the largest developing country, China’s rapid economic and social development has triggered fierce conflicts and confrontations between regional livelihood needs and ecological environment protection. Driven by multiple pressures such as environmental protection and anti-poverty efforts, The Chinese government stands at the height of “the fundamental plan concerning the sustainable development of the nation” [2], From the national strategy of ecological civilization construction and main function zoning to the actual implementation of high-quality development of the Yellow River basin and rural revitalization, large-scale ecological migration projects have been organized and implemented nationwide to actively explore and try to solve the above problems. In this context, accurately identifying the sustainable livelihoods of farmers in ecological resettlement areas is not only helpful for policy makers to deeply understand the actual contradictions of man-land relationship in resettlement areas, but also conducive to exploring countermeasures for the sustainable development of farmers’ livelihoods in resettlement areas, which is crucial for the construction of regional sustainable society.

The urgent situation of climate change, environmental crisis and deep poverty makes ecological migration research the focus of international organizations, governments and academic institutions. Foreign scholars have studied the theory and practice of ecological migration earlier, mainly focusing on the concept and classification of ecological migration [3,4], occurrence mechanism [5,6,7], the prediction of the subsequent development of migration [8,9,10], the relationship between ecological migration and environment [11,12,13] etc., and Chinese scholars pay more attention to the causes and purposes of ecological migration [14], sustainability and survival status of migrant society [15], social adaptation [16] and spatial evolution [17], ecological migration policy and management [18], economic benefits [19] and risk assessment [20], impact on the environment [21,22], etc. Over the past 30 years, with the continuous development of the practice of ecological migration, world scholars have conducted multi-level and all-round research on ecological migration from different disciplinary perspectives. It has formed a multi-disciplinary and multi-scale integrated research with multi-field and multi-scholar cooperation and has achieved fruitful results. As a typical region in China, the relationship between man and land is the most complex, the contradiction between man and land is the most prominent, and the economy–society–environment system is changing rapidly [23], The sustainable construction of the ecological economic corridor in the Yellow River basin is related to the transformation and development of the northern region and even the whole of China. The basic propositions of ecological environment protection, human land system coordination and sustainable development provide important strategic opportunities for the ecological protection and high-quality development of the Yellow River basin [24].

In terms of research methods, many evaluation methods have been developed, such as model evaluation, stakeholder evaluation and index evaluation. In summary, the model evaluation method and stakeholder evaluation method are simple to operate, but they have fewer factors to consider and have large errors in handling problems. Meanwhile, they are faced with challenges such as the lack of good validity and reliability of evaluation indicators and the lack of verification of evaluation results. On the other hand, most studies on sustainable livelihoods are conducted at the macro scale of countries, regions and cities, and few studies are conducted at the micro scale of ecological migrant groups or resettlement areas. In particular, the existing evaluation systems of conventional regions or groups are not applicable to the sustainable livelihood assessment of ecological migrants. Based on this, this paper based on the investigation of ecological resettlement areas in several regions of the upper reaches of the Yellow River, defines and grasps the essential attributes and core contradictions of the ecological resettlement area, and uses the analytic hierarchy process, Delphi method and expert judgment matrix to build an evaluation index system for the sustainable livelihood of farmers in the ecological resettlement area in the upper reaches of the Yellow River. From the perspective of human land relationship, the coupled coordination degree model was used to construct the evaluation model for the sustainable livelihood of farmers in the ecological resettlement area in the upper reaches of the Yellow River, and the Liujiaxia ecological resettlement area in Gansu Province was taken as an example to make an empirical test and modify the index system, so as to provide reference for the sustainable development of farmers’ livelihood in the Yellow River basin.

The rest of the paper is arranged as follows: Section 2 establishes a “three-way” systematic research framework for sustainable livelihood of farmers in ecological resettlement areas of the upper Yellow River. Section 3, based on the constructed “Ternary” system framework, proposes the evaluation system of farmers’ sustainable livelihoods in the ecological resettlement area of the upper Yellow River. Section 4 takes the ecological resettlement area of Liujiaxia Reservoir area as an example to verify and analyze the measure of farmers’ sustainable livelihood level. Section 5 puts forward the sustainable livelihood strategy of farmers in the ecological resettlement area of the upper Yellow River. Finally, Section 6 has a certain discussion, and Section 7 summarizes the research results.

## 2. The “Ternary” System Research Framework for the Sustainable Livelihood of Farmers in the Ecological Resettlement Area of the Upper Yellow River

### 2.1. The Policy Logic of Ecological Protection and High Quality Development in the Yellow River Basin

For thousands of years, frequent human activities have made the scale and intensity of ecosystem utilization and destruction in the Yellow River basin much higher than that in other regions [25]. Since the founding of the People’s Republic of China in 1949, the Yellow River Basin has experienced different stages of development, such as the inefficient development of deforestation, the rapid development of urbanization, and the green development of returning farmland to forest. In recent years, the contradiction between industrial upgrading, demand growth and ecological environment protection and development in the Yellow River basin has become increasingly fierce. The Chinese government has, to some extent, improved the man-land relationship and the ecological environment in the Yellow River basin through the policies and measures of “Yellow River control” and “Yellow River revitalization”, such as the western development, ecological civilization construction, water resources management reform and comprehensive watershed management [23]. However, the actual situation of rapid urbanization and ecological engineering construction still limits the overall industrial transformation and socio-economic development level of the Yellow River basin [26,27]. In 2018, ecological progress was written into the Constitution of the People’s Republic of China, realizing a high degree of unity between the Party’s position, the will of the state and the will of the people. A systematic, historic and long-term national strategic project under the guidance of ecological civilization [28]. How to “enrich the Yellow River” and “enrich the people” in the Yellow River basin has become a key issue to be solved urgently for ecological environmental protection and high-quality development in the Yellow River basin. The ecological resettlement area is a gathering place of population and economy, pursuing the trinity development of efficient economic growth, fair society and green and sustainable environment. However, for a long time, it is difficult for the resettlement area to realize the synchronous development of economy, society and environment. At present, the public has put forward new requirements for the development of resettlement areas, which should not only focus on economic growth, but also coordinate the three systems of economic development, social progress and environmental friendliness to achieve high-quality development as the new construction goal. In the three systems, environment is the foundation of development, economy is the support of development, and society is the key point of coordination between economy and environment. Therefore, how to coordinate the relationship between urban economic development, social progress and ecological environmental protection has become an important topic that the academic community pays common attention to, and the local government strives to solve.

### 2.2. The Construction of the “ Ternary” System Model of Farmers’ Sustainable Livelihood

With the rapid development of China’s economy, the competition of production, living and ecological space is becoming increasingly fierce [29]. Relying on the ecological protection and high-quality development strategy of the Yellow River basin under the guidance of the idea of ecological civilization construction, the “Ternary” system model of ecological resettlement area with the relationship between people and land as the core is constructed. The model is mainly composed of economic system characterized by agricultural production, social system characterized by farmers’ life, and environmental system characterized by rural ecology.

(1) Agricultural production: Economic system

The biggest development shortcomings of the Yellow River Basin are the weak economic foundation of urban and rural areas, the lack of livelihood security and development impetus. Due to the influence of various factors, the development of ecological resettlement areas in the Yellow River basin is lagging behind. How to activate the internal factors of ecological resettlement areas and realize the interaction between internal and external factors is the primary task to promote the economic development of the resettlement areas, with the continuous progress of urbanization in the Yellow River basin. With the attraction of regional advantage policies, through modern diversified industrial development, building an industrial system rich in regional characteristics of the Yellow River basin, expanding new agricultural and animal husbandry development models and other construction and exploration, the two-way free flow of urban and rural production factors and cross-border allocation have gradually become smooth. Large-scale human and financial capital investment in the Yellow River basin have gradually become the norm, and the advantages of urban and rural resources complement each other. The development trend of urban and rural industrial integration will be more obvious, and the development mode will be more modern and diversified.

(2) Peasant household life: Social system

As one of the regions with the most concentrated population, resource and environmental contradictions in China, various measures have driven the transformation of the relationship between people and land and the evolution of the urban and rural pattern in the Yellow River basin, whether it is the targeted poverty alleviation and rural revitalization strategy designed at the top level in recent years or the ecological migration assistance project at the micro level [30]. At present, the Yellow River basin, on the whole, in the stage of rapid development of urbanization, the regional residents of public service, living environment and health and safety aspects of the growing demand, in the traditional planting, animal husbandry for a living mass of the Yellow River basin area of agricultural population to cities and towns gathered, the gap between urban and rural development and residents’ living standard gap is narrowing, due to the acceleration of urban and rural integration development. The social development under the guidance of ecological protection and high-quality development in the Yellow River basin is the process of integrating the resources of the whole region, optimizing the spatial pattern of land, promoting the structural optimization and functional improvement of the rural regional system in the Yellow River basin, thus breaking the regional urban-rural dual opposition, and realizing the urban-rural integration and rural revitalization.

(3) Rural ecology: Environmental systems

The Yellow River basin is the key area covered by China’s major ecological protection and restoration projects, and the interaction between human activities and environmental change is strong [31]. It is a key area for studying man land relationship and man land system coupling process at home and abroad [32]. From the perspective of the environmental system space of the ecological resettlement area in the Yellow River basin, although farmers and herdsmen in the Yellow River basin have certain internal commonalities in their living environment and living conditions under the background of common culture and similar resource endowments, However, the contradiction between overall ecological protection and economic development, urbanization and rural revitalization is still prominent, The pressure of regional transformation and high-quality development is greater.

From the aspect of factors, the evolution of the “Ternary” system of economy, society and environment in the ecological resettlement area of the Yellow River Basin is a process of two-way free flow of all kinds of urban and rural factors such as people, land and production, gradually smooth cross-border allocation and reasonable allocation of public resources, from mutual restriction to coupling coordination of internal and external factors integration and optimization. From the structural perspective, the evolution of the “Ternary” system of economy, society and environment in the ecological resettlement area of the Yellow River basin is a process of complementary advantages between urban and rural areas and the coordinated development of different systems such as economy, society and environment. From the perspective of function, the evolution of the “three-way” system of economy, society and environment in the ecological resettlement area of the Yellow River Basin is a process of mining the multi-values of regional economy, society and environment based on the characteristics of the river basin, expanding and improving rural functions, gradually improving the living standards of residents and narrowing the urban-rural development gap.

**Figure 1:**
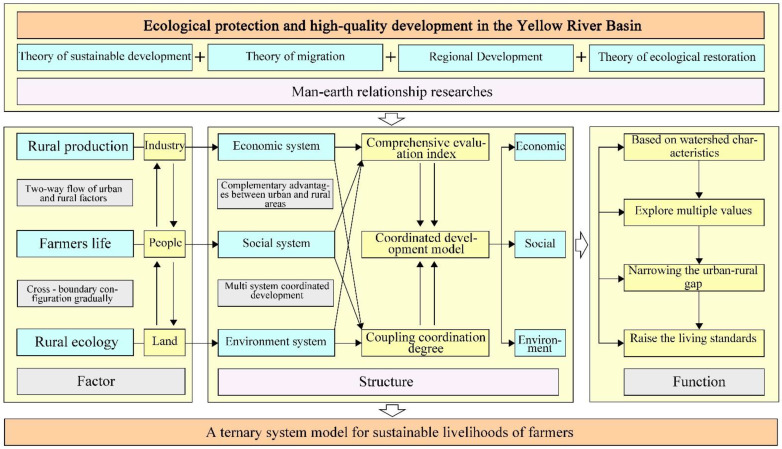
“Ternary” system model of farmers’ sustainable livelihood.

The issue of “agriculture, rural areas and farmers” is the concentrated embodiment of China’s basic national conditions and is the focus and difficulty of geography research for a long time. Through the integration, structural restructuring and functional optimization of various urban and rural system elements in the ecological resettlement area of the Yellow River Basin, the sustainable development of rural economy, society and environment in the ecological resettlement area of the Yellow River basin can be realized, to promote the effective implementation of national strategies such as new urbanization, agricultural modernization, urban-rural integration, and rural revitalization in the ecological resettlement area of the Yellow River basin. Based on this, this paper puts forward the basic logic of the construction of the sustainable livelihood evaluation system for farmers in the ecological resettlement area (Figure 1). That is to say, the sustainable livelihood of farmers in the ecological resettlement area is divided into three dimensions of economy, society and environment according to their basic connotation and contradiction focuses, and the development level of each subsystem is assessed separately, and then the overall sustainable livelihood level and type of ecological migrants are determined by examining the comprehensive evaluation index and the coupling relationship between subsystems.

## 3. The Construction of the Evaluation System for the Sustainable Livelihood of Farmers in the Ecological Resettlement Area of the Upper Yellow River Based on the “Ternary” System

### 3.1. Index Source

The United Nations Sustainable Development Goals (SDGs) are a result-oriented framework for sustainable development, mainly including 17 goals, 169 specific goals and the initial 231 specific indicators developed by the Inter-Agency Expert Group on SDGs (IAEG-SDGS) [33]. The aim is to encourage countries to use the framework to guide national planning, decision-making and investment decision-making, and regularly monitor and report on the progress of sustainable transformation from 2016 to 2030 [34]. It is the core concept and the axis principle that guides the economic and social development of all countries in the world. In September 2016, the Chinese government took the lead in releasing the National Program for China’s Implementation of the 2030 Agenda for Sustainable Development at the United Nations, which elaborated China’s specific action plan for implementing the SDGs. Linking ecological migration practices with SDGs will help improve the scientificity and globality of evaluation indicators, help realize China’s commitment to sustainable development, and promote the development of sustainable livelihoods of farmers in China’s ecological resettlement areas and the dissemination of China’s ecological migration experience. Due to the completion time of some targets in 2020, this study is mainly based on China’s Progress Report on the Implementation of the 2030 Agenda for Sustainable Development released by the Chinese government in September 2019 and the research group’s field visits to ecological resettlement areas in multiple regions in China. Based on the application time (2020–2030) of the comprehensive evaluation index system, the significance and pertinence of the evaluation indexes, the following types of indicators are eliminated in order: ① The targets that have been achieved in China in 2020, such as absolute poverty, universal coverage plan, electricity rate, road rate, etc. mean that only the specific targets that need to be further implemented in 2020–2030 remain. ② Indicators that are formulated and implemented by the Chinese government nationwide and do not show significant differences among ecological resettlement areas. ③ Indicators unrelated to the evaluation of sustainable livelihood of farmers in ecological resettlement areas, such as gender equality, AIDS incidence rate, and the proportion of women in the national parliament. ④ It is difficult for the research team to obtain relevant data, and at the same time, it is necessary to build indicators with less impact on the sustainable livelihood evaluation of farmers in China’s ecological resettlement areas, such as the number of victims of intentional homicide per 100,000 people, the number of researchers per million residents, etc.

### 3.2. Primary Selection of Indicators

The indicators of economic, social and environmental development subsystem of the sustainable livelihood evaluation system for farmers in ecological migration area mainly include three parts. The first part is based on indicators of economic, social and environmental dimensions in SDGs, the Country Programmer and the Progress Report, mainly including indicators with established methods and detectable indicators in the SDGs Global indicator framework and indicators with China’s statistical data in the UN database. The second part combines the sustainable livelihood approach [35] (SLA) proposed by department for international development (DFID) and the Livelihood framework proposed by united nations development programmer [36] (UNDP) creates a primary pool of indicators. The third part mainly refers to the indicator system established by researchers in various disciplines around the world. At present, there is no indicator system to evaluate the sustainable livelihood of farmers in ecological migration areas. Social system references include research indicators regarding ecological migration livelihood strategies [20], community space integration and reconstruction [37], dynamic evolution of ecological migration areas [38], influencing factors and intervention strategies of social relations reconstruction in ecological migration areas [39], social adaptation [40] and cultural adaptation [41] etc. Environmental system references include research indicators regarding resource endowment of resettlement areas [42], landscape structure [43] and evolution [44], land use change and ecological risk [45], resource carrying capacity [46] and ecological benefits [47] etc. After the primary selection of SDGs, after the preliminary selection of SDGs, the index system in the existing literature on sustainable livelihoods, livelihood vulnerability and capacity assessment and other related fields will be further sorted out, and several indicators will be added. Considering the SDGs primary index between the repetition and cross, and the docking with Chinese ecological immigration policy and practice, this article in SDGs and on the basis of “national plan” put forward the target, combined with relevant literature and field surveys, eventually form a corpus including 17 primary indexes and 99 secondary indicators of China’s ecological immigration in the evaluation of the sustainable livelihood of farmers primary index system (Table 1).

### 3.3. Index Excluding

#### 3.3.1. Expert Selection and Expert Positive Coefficient, Authority Coefficient

This paper uses the Delphi method to eliminate and screen the evaluation indicators. According to the Delphi method’s expert selection criteria, research groups were selected from 20 scholars: geography (4), anthropology (1), urban and Rural planning (3), history and culture (1), sociology (3), tourism management (3), ecology (5) and so on in many field, and in natural resources (3), housing (1) and agriculture (1) wait for multiple departments five government officials to form a team of experts. Among the 25 experts, there are 3 professors and 5 associate professors. The specific number of years for members to engage in ecological resettlement work is 7.5 (±2.5) years. The positive coefficient of experts is represented by the questionnaire recovery rate. The recovery rate of the two rounds of questionnaires was 100%, indicating that the experts paid high attention to the ecological migration research of the research group. The judgment coefficient refers to the judgment basis of experts on the importance of each indicator of the index system (Table 2), which includes four categories: theoretical analysis, practical experience, peer understanding and intuition. The expert authority coefficient is expressed by the arithmetic mean of the familiarity coefficient and the judgment coefficient. The familiarity coefficient refers to the experts’ familiarity with the contents related to ecological migration (0 for unfamiliar, 0.25 for less familiar, 0.50 for general, 0.75 for more familiar, 1.00 for very familiar). In this paper, the familiar coefficient of consulting experts is 0.83, the judgment coefficient is 0.77, and the expert authority coefficient is 0.8.

#### 3.3.2. Expert Consultation Process and Results

Using the Likert five-level scale, the research team conducted the first round of inquiry letter from 15 to 25 April 2022, according to the importance, and invited experts to score the primary indicators of the three subsystems of economy, society and environment according to the importance. After the first round of inquiry, the average was calculated based on the results of expert ratings (P), first quartile (Q1), third quartile (Q3), standard deviation (SD), and coefficient of variation (CV). Seven indicators with low importance (Q1 < 3, Q3 < 4 and P < 3) were excluded. They are respectively the brand awareness of the economic subsystem, the kindergarten enrollment rate of the social subsystem, green education, women’s participation in management, and the village greening degree, forest coverage and sewage and waste treatment of the environmental subsystem. After the first round of elimination, there were six indicators with low coordination (SD > 1, CV > 0.25). From 5 to 15 May 2022, the research group organized the second round of inquiry letters. On the basis of feeding back the mean of the indicators scored by the experts in the first round to the experts, the experts were invited to score the revised indicators again. Based on the results of the second polling letter, six indicators were removed, including tourism business houses and local employment opportunities in the economic subsystem, neighborhood relations and cultural ownership in the social subsystem, and sanitary toilet penetration and communication penetration in the environmental subsystem. After two rounds of evaluation by experts, the remaining indicators meet the requirements in terms of importance and coordination. Further, Kendall’s Wa test was carried out on the expert scoring results, *p* = 0.000 < 0.05, It proves that there is significant consistency in expert scores and the prediction results are reliable. Finally, a sustainable livelihood evaluation system of farmers in ecological migration resettlement area with 85 indicators covering three sub-systems of economy, society and environment was formed (Table 3).

### 3.4. Data Processing

#### 3.4.1. Construction of Judgment Matrix and Consistency Check

In the subsystems of economic, social and environmental dimensions of the sustainable livelihood evaluation system for farmers in ecological resettlement areas, the effects and influences of each indicator are different, so it is necessary to assign weights to each indicator. Considering the actual situation of ecological migration and the complexity of the indicator system, this paper adopts the analytic hierarchy process (AHP) in the subjective weighting method. After constructing the hierarchical structure model, it is divided into two stages to obtain the weight of indicators at each level. The first stage is to obtain the weight of the first-level indicator layer through the expert judgment matrix, and the acquisition process is divided into three steps. Firstly, in order to reduce the distortion of evaluation results caused by human factors as much as possible, this paper uses Deplphi method to solicit expert opinions and invite 25 experts to construct pairwise comparison judgment matrix between first-level indicators to determine the weight of each indicator. The weight is calculated using the following formula:

(1) $$L_{i}=\sum^{\;}x_{ij}/z$$

Among them, *L_i_* ∈ (0, 1), $x_{ij}$∈ (0, 1), $\sum^{\;}L_{i}$ = 1. *L_i_* is the index weight of *i*, *z L_i_* is the index weight of *i*, *z* is the number of experts, $x_{ij}$ is the scoring value of index *i* by the *j*th expert. Since *L_i_* is an integer in the judgment matrix, the pairwise comparison between judgment factors will use the integer to express the weight value by rounding [56]. According to the weight calculation formula, the evaluation of each expert is integrated, and the judgment matrix of the first-level index in the three subsystems of economy, society and environment is determined on this basis. Finally, applying Yaahp12.1 software to the expert group, it was decided to calculate the weight results of the first-level index layer and carry out consistency test to obtain *C_i_* = 0.0925 < 0.1. The consistency test of the judgment matrix passed, that is, the judgment of the primary indicators of sustainable livelihood of farmers in the ecological resettlement area was consistent, and the weights obtained were reliable. The second stage is to further allocate weight to secondary indicators according to the average value of experts’ scores (Table 3).

#### 3.4.2. Data Sourcing and Standardization

The sustainable livelihood assessment of farmers in ecological resettlement areas in the upper reaches of the Yellow River mainly obtains data in two ways. ① The socio-economic development, resource and environment data mainly come from the ecological migration related data and statistical yearbooks published by the local village committee, town government and other government agencies at all levels. The resource and environment data mainly come from the land use data of the third National Land Survey. ② Participatory Rural Assessment method (PRA): questionnaire survey, structured interview, basic information sampling and other methods were used to obtain case study and analysis data of farmers in ecological migration areas.

The sustainable livelihood evaluation of farmers in ecological resettlement area is an index evaluation of administrative villages or natural villages in the resettlement area, which actually involves two aspects: research and management. Therefore, for further scientific research, the construction of evaluation index system is not only required to be scientific and rigorous. On the other hand, the proposal of the evaluation index system must meet the requirements of the management and evaluation of ecological migration by departments at all levels, so we must also pay attention to the practicality and operability of the index system. In terms of data sources, there are many contradictions and problems in the data related to ecological migration, such as lack of statistical data, strong confidentiality, low credibility, difficult data acquisition, and complex sources. In terms of data processing, there are a series of problems, such as the difference of magnitude and direction between different original data, and the difficulty of cross-year and cross-region comparison. Previous studies on the index system often used range standardization to process the original data, so as to make different indicators comparable across regions and years and eliminate the differences in magnitude and direction among the original data. The range standardization formula is as follows:

(2) $$N_{\mathrm{ij}}=\frac{N_{ij}-\min\left(N_{ij}\right)}{\max\left(N_{ij}\right)-\min\left(N_{ij}\right)}\;\mathrm{Positive}\;\mathrm{indicator}$$

(3) $$N_{\mathrm{ij}}=\frac{\max\left(N_{ij}\right)-N_{ij}}{\max\left(N_{ij}\right)-\min\left(N_{ij}\right)}\;\mathrm{contrary}\;\mathrm{indicator}$$

where: *N_ij_* is the standardized value of the *j*th index of system *i*; *N_ij_* is the original value of the *j*th index of system *i*; max (*N_ij_*) and min (*N_ij_*) are the maximum and minimum values of the *j*th index of system *i*, respectively. Min-max standardization was used to normalize the evaluation index data, and zero value would appear after standardization. The zero-value index was processed by translation and Translation deals with zero value indicators.

### 3.5. Comprehensive Calculation

#### 3.5.1. Calculation of Comprehensive Evaluation Index

The economic, social and environmental subsystems in the evaluation system of sustainable livelihood of farmers in the ecological resettlement area are calculated using the multi-objective linear weighting function method, and its function expression formula is:

(4) $$W=\sum_{i=1}^{m}\left(L_{i}\times E_{i}\right)$$

where: *W* is the assessment value of economic, social or environmental system; *L_i_* and *E_i_* are the standardized value and weight of the *i*th index, respectively.

The comprehensive evaluation index of sustainable livelihood of farmers in ecological resettlement areas in the upper reaches of the Yellow River is *K*. In order to reflect the interrelationships among subsystems and avoid reducing the value range of *K* due to geometric weighting calculation, the comprehensive evaluation index *K* in this study is calculated by arithmetic weighting, and its calculation formula is as follows [57]:

(5) $$K=\sum_{i=1}^{m}\left(Q_{i}\times W_{i}\right),\;\sum_{i=1}^{m}Q_{i}=1$$

*W_i_* and *Q_i_* are the values and weights of the *i*th subsystem respectively. In this paper, *n* = 3, and economic, social and environmental systems are equally important in the evaluation process, so *Q_economy_* = *Q_society_* = *Q_environment_* and $\;Q_{1}+Q_{2}+Q_{3}=1$, namely:

(6) $$K=Q_{\mathrm{economy}}\times \;W_{\mathrm{economy}}+Q_{\mathrm{society}}\times \;W_{\mathrm{society}}+Q_{\mathrm{environment}}\times \;W_{\mathrm{environment}}=\;\frac{1}{3}(W_{\mathrm{economy}}+W_{\mathrm{society}}+W_{\mathrm{environment}})$$

The comprehensive evaluation index *K* is distributed in [0, 1], which can be used to evaluate the comprehensive level of sustainable livelihoods of farmers in China’s ecological resettlement areas. The evaluation criteria are shown in Table 4.

#### 3.5.2. Coordinated Development Calculation

As a multi-dimensional complex giant system, ecological migration involves multiple internal coupling relationships among different systems, such as economy, society and environment. Therefore, with the help of coupling effect and coupling coordination degree model, the research uses coupling degree to explain the mutual relationship between subsystems and uses coordination development degree to comprehensively evaluate and study the whole system. Currently, the widely used normative formula of coupling degree model is [58]:

(7) $$R={\left[\frac{\prod_{i=1}^{n}W_{i}}{{\left(\frac{1}{n}\sum_{i=1}^{n}W_{i}\right)}^{n}}\right]}^{\frac{1}{n}}$$

where: *n* is the number of subsystems; *W_i_* ∈ [0, 1] is the value of each subsystem, and the coupling degree *R* ∈ [0, 1]. The larger the *R* value is, the smaller the dispersion degree between subsystems is, and the higher the coupling degree is. On the contrary, the coupling degree is lower. Coupling coordination degree is a comprehensive indicator of coupling degree and development level. It can reflect the coordination relationship among the three subsystems of economy, society and environment, and also reflect its development level. The coordinated development model of “economy—society—environment” system constructed in this paper is as follows:

(8) $$J=\sqrt{R\times K}$$

### 3.6. Spatial Distribution Differences

When some variables have potential interdependence among the observed data in the same distribution area, the spatial autocorrelation method can be used for spatial analysis. A spatial autocorrelation model was further constructed to analyze the spatial aggregation characteristics of the sustainable livelihood level of farmers in the case sites, mainly including global spatial autocorrelation analysis and local spatial autocorrelation analysis, which were characterized by Moran’s *I* index and LISA graph (Local indicators of Spatial association). The formula is:

(9) $$I=\frac{N\sum^{\;}i\sum^{\;}jY_{ij}\left(X_{i}-\bar{X}\right)\left(X_{j}-\bar{X}\right)}{\left(\sum^{\;}i\sum_{j}Y_{ij}\right)\sum_{i=1}^{n}{\left(X_{j}-\bar{X}\right)}^{2}}$$

(10) $$I=\frac{X_{j}-\bar{X}}{S_{x}^{2}}\sum_{j}\left[Y_{ij}{\left(X_{j}-\bar{X}\right)}^{2}\right]\;\;$$

(11) $$\;\;\;\;S_{x}^{2}=\frac{\sum_{j}Y_{ij}{\left(X_{j}-\bar{X}\right)}^{2}}{n}\;$$

## 4. Measurement of the Sustainable Livelihood Level of Farmers in the Ecological Resettlement Area of Liujiaxia Reservoir Area

### 4.1. Study Area and the Data Sources

#### 4.1.1. Overview of the Study Area

**Figure 2:**
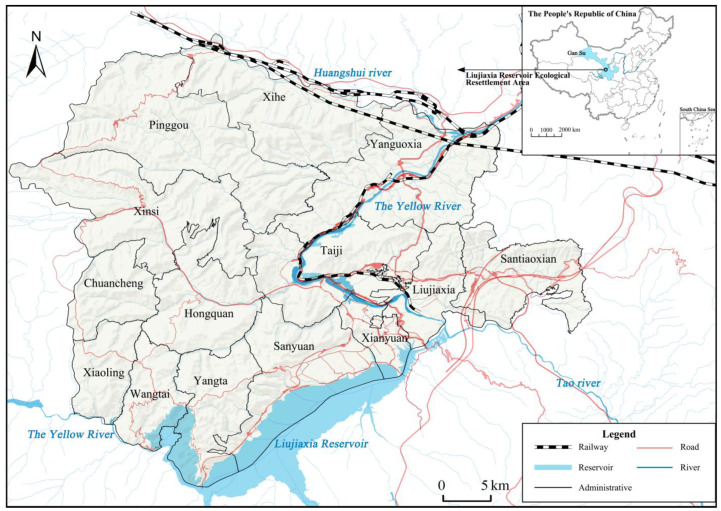
Location of ecological resettlement area in Liujiaxia Reservoir Area.

The Yellow River is the mother river of the Chinese nation, giving birth to the ancient and great Chinese civilization. As one of the rivers with the highest sediment content in the world, and the most difficult to control and the most serious water damage, the Yellow River is faced with complex problems and difficulties such as water resource shortage, fragile ecological environment, sharp contradiction between man and land, and relatively backward economic development. The contradiction between development and protection is very prominent. As a typical ecological resettlement area in the upper reaches of the Yellow River, the practice of ecological migration in Liujiaxia Reservoir Area began in the construction period of the reservoir area (including Liujiaxia, Yanguo Gorge, Bapanxia and other hydropower stations). As the first megawatt hydropower station in Asia that China has independently surveyed, designed and built, The Liujiaxia Reservoir Area, represented by the Liujiaxia Hydropower Station, plays an important role in flood control, storage, shipping and irrigation in the Yellow River Basin, and provides power support for the economic construction and development of Northwest China and even the whole country. Due to historical legacy, policy change, resource endowment, benefit distribution and other factors, the ecological migration problem in Liujiaxia reservoir area has not been properly solved. The ecological resettlement area of Liujiaxia reservoir area involved in this study has a total of 66,669 people in 14 townships. At present, the overall ecological environment of the case is fragile, water conservation function is weakened, soil and water loss is serious, and the ecological system is gradually degraded. Economic development is mainly based on the planting and breeding industry. The industry is single and has not yet formed a complete industrial chain. The overall level of social development is relatively backward, and it is one of the poverty-stricken counties that China focuses on supporting (Figure 2). As a typical migration area in the upper reaches of the Yellow River, the ecological resettlement area in the Liujiaxia Reservoir area can better reflect the sustainable livelihood level of farmers in the upper reaches of the Yellow River. Therefore, it is of great theoretical value and practical significance to study the measurement and spatial differentiation characteristics of farmers’ livelihood capital in this area.

#### 4.1.2. Data Sources

On the basis of the historical reality of ecological migration in Liujianxia Reservoir area, this paper selects 14 townships involved in ecological migration resettlement in Yongjing County as the empirical test objects of the sustainable livelihood evaluation system of farmers in the ecological migration resettlement area in the upper reaches of the Yellow River (Table 5).

First of all, the team learned about the social and economic development of the case through the official websites of governments at all levels, statistical yearbooks and other channels, and preliminarily designed the questionnaire and interview outline based on the existing literature. Secondly, based on the modification and improvement of the questionnaire and interview outline according to the pre-investigation, the research group conducted three sample surveys and field visits in the above case sites from September 2021 to April 2022. A stratified random sampling method was adopted to randomly select 40~60 households in each administrative village for household entry. Face-to-face interviews, questionnaire surveys and observation were mainly adopted. The investigators were divided into 10 groups with 2 people in each group and the survey time of each household was about 20~30 min. Excluding the rural households who go out all year round but still have household registration in the area, and some invalid questionnaires, a total of 1248 questionnaires were issued according to the actual population status of the 14 towns in the case, and 1116 valid questionnaires were finally recovered, accounting for 89.42% of the total number of questionnaires. Among them, 56.65% were unregistered and 43.35% were registered. The characteristics of the surveyed households are shown in Table 6.

The main subjects of the questionnaire survey are the groups that pay high attention to sustainable livelihood, namely the rural households in the resettlement area. The questionnaire survey and interview mainly include three parts: first, the overall basic information about the sustainable livelihood of the rural households; second, the implementation of resettlement policies and welfare; the third is the basic information of peasant households, including family structure, the number of labor force, health status, education level, etc. In addition, in June 2022, the research team made two additional visits to the case sites and returned visits to some representative farmers to further improve the relevant data on sustainable livelihoods of farmers in the case sites.

The data involved in the empirical study mainly include three parts: ecological migration survey data, social and economic development data and resources and environment data conducted by the research group in Liujiaxia Reservoir area. The ecological migration survey data were collected by the research team. The socio-economic development data of Liujiaxia Reservoir area mainly include population data, industrial data and GDP data obtained from the case area statistical yearbook, and the resource and environmental data mainly include land use data from the third National Land survey. Finally, in consideration of Ethical consideration, we collect data anonymously in the process of investigation.

### 4.2. Evaluation of Sustainable Livelihood Level of Farmers in Liujiaxia Reservoir Area

#### 4.2.1. Spatial Distribution Difference

**Figure 3:**
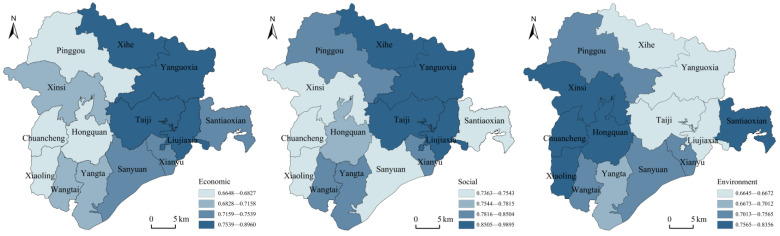
Evaluation results of sustainable livelihood subsystem of ecological migration area in Liujiaxia Reservoir area.

Overall, the 14 townships in the Liujiaxia Ecological Resettlement Area have relatively good sustainable livelihood ratings, and there are large differences among different towns in each system. The economic subsystem index is 0.8837~0.6648, the social subsystem index is 0.9824~0.7363, and the environmental subsystem index is 0.8356~0.6645. The overall level of economic subsystem and social subsystem is relatively high, and the overall level of environmental subsystem is relatively low (Figure 3).

The comprehensive evaluation index of sustainable livelihood of farmers ranged from 0.8437~0.7435, among which the comprehensive evaluation index of sustainable livelihood of Liujiaxia, Yanjiaoxia, Taiji and Xihe towns was excellent, and the comprehensive evaluation index of sustainable livelihood of other towns was good. The coordinated development degree J ranged from 0.7544~0.7119, and all townships were classified as intermediate coordinated development (Table 7).

#### 4.2.2. Spatial Distribution Differences

Through the global autocorrelation model, the global Moran’s *I* value of the case farmers’ sustainable livelihood capital index is obtained (Table 8), and the distribution of different systems of the case farmers’ sustainable livelihood is explored. Moran’s *I* values of economic, social and environmental systems are all greater than 0, among which, the normal statistics Z value and significance level test value of Moran’s *I* of economic, social and environmental systems are both greater than the critical Z value (1.96) under the confidence level of 0.05, indicating that the economic, social and environmental systems of farmers’ sustainable livelihood in the study area show significant and positive spatial autocorrelation characteristics in the overall situation, that is, the distribution has significant spatial dependence, and it has obvious gathering characteristics.

**Figure 4:**
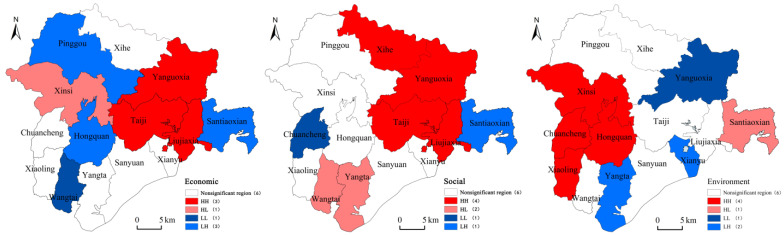
LISA significance distribution of sustainable livelihood in ecological migration area in Liujiaxia Reservoir area.

The sustainable livelihood level of farmers in the ecological resettlement area of Liujiaxia Reservoir Area has a strong coupling with its geographical location. The overall phenomenon is that the closer to the Yellow River, the higher the level of sustainable livelihoods, and the local phenomenon is a stable two-way agglomeration of high and low value areas, with obvious differences between high and low values. Since the Moran scatter plot does not give an indicator of the significance level, the LISA value is calculated to further explore the local variation characteristics of the sustainable livelihood level of farmers in different systems in the case site. Geoda-1.8.12 software was used to characterize the local Moran’s *I* value of each unit and its corresponding significance level to obtain the local Lisa of the sustainable livelihood level of farmers in the case area (Figure 4). From the perspective of local change characteristics, the sustainable livelihood level of farmers shows a stable two-way agglomeration of high and low values, that is, high value and high value (H-H), low value and low value (L-L), with obvious spatial differences between high and low values. H-H, L-L agglomeration indicates that the high value and low value areas of farmers’ sustainable livelihood level are spatially similar to their adjacent regions, and the relationship between regions shows a significant hierarchical diffusion structure. On the other hand, the case has formed a spatial agglomeration pattern dominated by different systems. Among them, the economic system dominates the low-value agglomeration, and the social and environmental system dominates the high-value agglomeration. The economic and social systems are mainly distributed in Taiji, Liujiaxia, Yanguoxia and other townships in the eastern part of the case site. The area is adjacent to Lanzhou City, and the economic conditions are relatively good. The environmental system is mainly distributed in Xinsi, Hongquan, Yangta and other townships in the western region of the case site. The livelihood of farmers is mainly based on traditional planting and animal husbandry, and the natural ecological environment in the region is relatively good. Overall, the sustainable livelihood level of farmers in the ecological migration resettlement area of Liujiaxia Reservoir area has a certain spatial aggregation, and the analysis results of spatial autocorrelation are basically consistent with the evaluation results of the sustainable livelihood level of farmers.

#### 4.2.3. Analysis on the Influencing Factors of Sustainable Development of Ecological Migration

Through the evaluation of the sustainable livelihood of 14 townships in the ecological resettlement area of Liujiaxia Reservoir Area, it can be seen that the sustainable livelihood level in different ecological resettlement areas is significantly different, the influencing factors are analyzed from three levels: indicator weight (coefficient), actual development (independent variable), and coordination relationship (dependent variable).

(1) The index weight of each subsystem formed by the expert judgment matrix can be regarded as the stability coefficient of the sustainable livelihood evaluation of farmers in the ecological resettlement area. In the first-level index layer, the main influencing factors of the economic development subsystem are: physical capital status (0.4689), financial capital status (0.2316), development conditions (0.1380), risk resistance (0.0681), tourism capital status (0.0552) and economic benefits (0.0405). The main influencing factors of the social development subsystem are: human capital status (0.3711), social capital status (0.3645), social governance level (0.1500), social construction level (0.1041), social equity (0.0774), cultural capital status (0.0375), environmental adaptation level (0.1233) and climate change and impact (0.0462). In the second-level indicator layer, the top five single-indicator factors in the impact level of the economic development subsystem are family fixed assets (0.1320), family living facilities (0.1209), homestead area (0.1135), number of livestock breeding (0.1024) and family deposit (0.0592). The top five single indicator factors of social development subsystem impact level are social support (0.0793), skills training opportunities (0.0757), location advantage (0.0735), social welfare (0.0735), family population size (0.0683) and non-agricultural livelihood level (0.0683). The top five single index factors of environmental development subsystem impact level are grassland resources (0.1205), water resources (0.1217), cultivated land resources (0.0928), forest resources (0.0892), environmental satisfaction rate (0.0347), and traffic satisfaction (0.0347). From the overall weight of indicators, physical capital, natural capital and human capital are the indicators with the largest weight, indicating that experts believe that these three indicators have the greatest impact on the sustainable livelihood of farmers.

(2) The actual development difference of each evaluation index in the ecological resettlement area is the main factor affecting the evaluation of sustainable livelihood level in the ecological resettlement area, which can be understood as the independent variable of sustainable livelihood development and evolution in the ecological resettlement area. The evaluation of 14 townships shows that the internal advantages and disadvantages of the subsystems of economic, social and environmental development are different among different evaluation objects. For example, Liujiaxia Town has the best development of physical capital and financial capital, while Chuanchen town has the best evaluation of natural capital and environmental carrying capacity.

(3) The coordination of dependent variables among the three subsystems of economy, society and environment is an important factor affecting the type of coordinated development of ecological resettlement areas. The sustainable livelihood level calculated by the comprehensive development index cannot fully reflect the actual sustainable livelihood level of farmers in the ecological resettlement area by only considering the arithmetic average of the three subsystems. Therefore, the coordinated relationship among the dependent variables among the economic, social and environmental subsystems significantly affects the evaluation of the final coordinated development type, which can effectively make up for the shortcomings of only calculating the comprehensive evaluation index, and more energetically reflect the core idea of sustainable livelihood of the coordinated development of the economy, society and environment in the ecological resettlement area.

## 5. Responses to Sustainable Livelihood Strategies of Farmers in Ecological Resettlement Areas in the Upper Reaches of the Yellow River

### 5.1. Economic Dimension Development Strategy of Sustainable Livelihood of Peasant Households in Ecological Resettlement Area of the Upper Yellow River

Because of its unique regional system, the Yellow River Basin determines that to achieve compatibility between regional industrial development and ecological protection, and to increase the income of farmers and herdsmen in resettlement areas, it is necessary to promote industrial development and improve overall social economic benefits. At present, the livelihood of farmers in most areas of the Yellow River Basin is still dominated by traditional planting and animal husbandry, and a series of problems such as insufficient industrial linkage, urgent need to optimize industrial layout, and lack of characteristic regional brands are prominent. In response to the above problems, it is necessary to give full play to the advantages of resources, location, culture, etc., with the goal of promoting the development of regional industries, focusing on unique resources and advantageous industries, to build an industrial system with characteristics of the Yellow River Basin, expand a new development model of agriculture and animal husbandry, and create a modern economic development model with modern agriculture and animal husbandry and human resource output as the core; at the same time, modern enterprises are introduced into the resettlement area to form a modern cooperative management model, and to continuously improve the level of organization and modernization. The versatility of the development of agriculture and animal husbandry is utilized to promote the deep integration of agriculture and animal husbandry with tourism, culture, education, service and other industries.

### 5.2. Social Dimension Development Strategy for Sustainable Livelihood of Farmers in the Ecological Resettlement Area in the Upper Reaches of the Yellow River

The development strategy of the sustainable livelihood social system for farmers in the ecological resettlement area in the upper reaches of the Yellow River is mainly composed of three aspects: social life, social governance and social culture. Social life is mainly about the development of employment structure and way of life. Driven by the high-quality development of the Yellow River Basin, the optimization of economic structure and industrial development, farmers in the resettlement area have changed from traditional planting, animal husbandry or migrant workers to planting and farming with modern characteristics. Breeding provides cultural tourism and human resources services. The change of the employment structure and more diversified and modern lifestyles of farmers in the resettlement area, improve the living environment, realize the full coverage of modern public facilities, and greatly improve the quality of life of the farmers in the resettlement area is promoted. In terms of social governance, due to the long-term blockade and poverty in the upper reaches of the Yellow River, there are many problems such as serious waiting and thinking. By exploring and improving the model of “cooperatives + characteristic industries + farmers”, the current weak grass-roots governance will be transformed into the administrative governance of the village branch and the two committees, supplemented by the people’s self-governance, stimulate the “internal power” governance of the resettlement area, and clarify the village collective in the resettlement area, the ownership of property rights, the cultivation of business entities, and the development of collective economy. According to the actual situation of the resettlement area, village rules and regulations to improve the quality of social governance are formulated. At the social and cultural level, it is intended explore and innovate the characteristic culture of the Yellow River Basin and integrate into the development of the regional cultural tourism industry. Social culture pays more attention to the combination of regional culture and modern culture, inheriting and innovating the connotation of rural culture.

### 5.3. Development Strategy of Sustainable Livelihood Environment Dimension for Farmers in Ecological Resettlement Areas in the Upper Reaches of the Yellow River

Based on a comprehensive analysis of the interaction and coordination between the ecosystem and human society in the Yellow River Basin, it is urgent to scientifically coordinate soil and water conservation, ecological construction and the development of efficient dry farming in the Yellow River Basin by integrating the natural resources elements of the whole region, optimizing the spatial pattern of national land, strengthening regional ecological construction, environmental protection and comprehensive management of water, soil and gas, highlighting the characteristics of high-quality development and economic and social transformation of the basin, and promoting the construction of ecological co-governance in the basin.

## 6. Discussion

The ecological resettlement area is an important bearing area for human society to solve the ecological crisis and coordinate the relationship between man and nature. It is of great value for the sound development of regional economy, society and environment to build an evaluation system for the sustainable livelihood of farmers in the ecological resettlement area and objectively evaluate the livelihood status and sustainable livelihood level of farmers in the resettlement area. This paper makes contributions at the following levels:

(1) Construction of evaluation system.

The sustainable livelihood evaluation system of farmers in the ecological resettlement area of the upper reaches of the Yellow River constructed in this paper can more accurately and comprehensively assess the sustainable livelihood level of farmers in the ecological resettlement area of the upper reaches of the Yellow River through the rigorous construction process of the index system and the introduction of the coupling coordination degree model. At the same time, the screening and determination of multidimensional evaluation indicators lay the foundation for the future research and evaluation of different scales of resettlement areas. It can not only carry out continuous tracking research on specific villages or regions, but also make comparative research and internal difference analysis on the development level of ecological resettlement areas at different geographical scales from villages to regions. This is an important promotion of the research paradigm on the livelihood of farmers in ecological resettlement areas.

(2) The use of research methods.

The standard system of data sources, indicator scoring and multi-level results division built in this study can not only promote the research of coupling degree and traditional development evaluation paradigm, but also improve the practicability and operability of the evaluation system by solving the problems of data volatility and incomparability caused by the current standardized processing. It is widely used in the evaluation and guidance of the sustainable livelihood level of farmers in the ecological resettlement area.

(3) Model empirical test.

In order to verify whether the evaluation system proposed in this paper can effectively reflect the sustainable livelihood level of farmers in the resettlement area and identify the differences among the evaluation objects, this paper selects a small scale and the same type of ecological resettlement area in the Liujiaxia Reservoir area for empirical test. The ecological migration project in Liujiaxia Reservoir Area, as the earliest large-scale ecological migration practice in China, has been highly valued by both the national and local governments. The evolution and development of different dimensions of economy, society and environment in this region are in line with the ecological migration practice in China, which has a high representative significance. Based on the sustainable livelihood evaluation of 14 townships in the ecological resettlement of Liujiaxia Reservoir area, it can be judged that the validity of the sustainable livelihood evaluation system for farmers in the ecological resettlement area constructed in this paper is relatively good, and the evaluation results have significant discriminant validity and microscopic differences. explanatory power. Comparing the evaluation results with the field investigation and visits and feeding back the evaluation conclusions to the 25 expert groups concerned with ecological resettlement issues, it is generally believed that the evaluation system can objectively reflect the actual livelihood status and sustainable development of farmers in the ecological resettlement areas in the upper reaches of the Yellow River. subsistence level.

On the other hand, the evaluation system constructed in this paper still needs some empirical correction in the following aspects. ① Validity test. Due to space limitations, this paper only selects Liujiaxia Reservoir Area as the resettlement area for empirical test. Although this area is highly representative, as far as the upper reaches of the Yellow River is concerned, the resource endowment, formation history, development characteristics and levels of resettlement areas in different regions are different. ② Indicators are simplified. At present, there are a large number of second-level indicators in the index system, which makes it difficult to collect and organize data. In the future, it is necessary to collect a large number of peasant households’ data in the resettlement area and conduct correlation analysis on the indicators, so as to further eliminate the indicators with higher correlation coefficients, so as to build a more refined evaluation system. ③ Score correction. At present, the basic data of the secondary indicator scoring are obtained by the research team through field research, interviews, questionnaire sorting and public data sorting and analysis. Considering that the economic, social and environmental development level of Gansu Province is at the level of the middle reaches of the upper reaches of the Yellow River, the indicator scoring standard needs to be revised in the future according to the empirical data of other resettlement areas in the upper reaches of the Yellow River.

Sustainable livelihood is a complex system with environmental, economic and social dimensions. With the drastic changes of resources and environment, the intensification of economic globalization, the expansion of population migration scale and the rapid advancement of urbanization, the disturbances to sustainable livelihood will become more diversified and cross-scale, and livelihoods will become more dynamic and complex. The sustainable livelihood of farmers is always closely related to specific economy, society and environment. At present, sustainable livelihood research focuses on the consideration of single economic, social or environmental factors [59,60].

For example, Woyesa et al. analyzed the impact of social and environmental changes on sustainable livelihoods [61]. The environmental livelihood security framework proposed by Eloise M Biggs et al. [62], Sherbinin et al. revealed the multipath relationship between environmental factors and sustainable livelihoods [63]. Understanding the interaction between sustainable livelihoods of rural households and the economy, society and environment is critical to promoting sustainable development. In the future, we should pay attention to the interaction process and mechanism between sustainable livelihoods and economy, society and environment, conduct research on livelihood security, vulnerability and spatial differentiation of sustainable livelihoods from different perspectives of economy, society and environment, and pay attention to the simulation and prediction of the evolution trend of sustainable livelihoods under different scenarios.

## 7. Conclusions

The main research conclusions of this paper are as follows: First, on the basis of analyzing the policy logic of ecological protection and high-quality development in the Yellow River Basin, this paper constructs a “Ternary” system model of sustainable livelihood of farmers in the ecological resettlement area in the upper reaches of the Yellow River, and proposes that the interrelationship between the three basic latitudes of economy, society and environment is the key to the sustainable livelihood of farmers in the ecological resettlement area. Secondly, based on the research model, an evaluation system of farmers’ sustainable livelihoods including economic, social and environmental dimensions is constructed by using AHP and Delphi method. The comprehensive evaluation index *K* was used to evaluate the sustainable livelihood level of ecological migrants. At the same time, the coupling coordination degree R is introduced to construct the coordinated development degree model *J* of “economy-society-environment” system. Then, based on the questionnaire survey and in-depth interview data in the ecological resettlement area of Liujiaxia Reservoir area in the upper reaches of the Yellow River Basin, the sustainable livelihood status and spatial distribution differences of 14 towns in the region were evaluated, and the good validity of the evaluation system was verified. Finally, in view of the economic, social and environmental dimensions, the sustainable livelihood strategy of farmers in the ecological resettlement area of the upper Yellow River was proposed, and the index system was further modified.

It is one of the important ways to understand the reality of social poverty to measure the sustainable livelihood level of farmers in the ecological resettlement area. In the future, attention should be paid not only to the influencing process and influencing mechanism of various factors on the sustainable livelihood level of farmers, but also to the interaction relationship between various factors, so as to reveal the degree and path of their effects on the sustainable livelihood level of farmers. At the same time, intervention research on sustainable livelihood policies should be strengthened, and the effectiveness of various policies should be reasonably evaluated.

## Figures and Tables

**Table 1:** Primary indicator system for sustainable livelihood of farmers in the ecological resettlement area of the Yellow River basin [48,49,50,51,52,53,54,55].

First-Level Indicator	Secondary Indicators	Nature	Source	First-Level Indicator	Secondary Indicators	Nature	Source
Economic subsystem				Environment subsystem			
Economic benefit	Agricultural income share	−	I/II/III	Natural capital status	Cultivated land resources	+	I/II/III
	Engel coefficient	−	IV		Forest Resources	+	I/II/III
	Proportion of expenditure on culture, education and entertainment services	+	IV		Grassland resources	+	I/II/III
	Private car ownership	+	IV [48]		Water resources	+	I/II/III
Development condition	Characteristic industry development	+	II/IV [48]	Environmental bearing condition	The population density	+	I/IV [51]
	Industrial diversification	+	IV [49]		Ecological carrying capacity	+	IV [51]
	Local employment opportunities	+	I/II/III		Per capita cultivated land area	+	IV
	Livelihood diversity index	+	I/II/III		Per capita water resources	+	I/II/III
Resistance to risk	Household debt ratio	−	I/II		Environmental protection investment intensity	+	I/II/III
	Labor employment rate	+	I/II/III		Ecological footprint	+	I/IV [52]
	Labor insurance coverage	+	I/II/III		Forest coverage	+	I/II/III
	Total grain production	+	I/II	Climate change and impacts	Impact of desertification	−	I/II/III
	GDP per capita	+	I/II/III		Incidence of meteorological disasters	−	I/II/III
	Economic growth rate	+	I/II/III		Disaster prevention capacity	−	I/II/III
	Urban-rural income gap	−	I/II		Impact of desertification	+	I/II/III
	Community gap between rich and poor	−	I/II	Environment adaptation level	Satisfaction rate of agricultural land quality	+	I/II/III
Physical capital status	Household fixed assets	+	Ⅵ		Environmental satisfaction rate	+	I/II
	Area of homestead	+	V		Traffic satisfaction	+	I/II
	Livestock breeding	+	I/II/III		Satisfaction with community security	+	I/II/III
	Family living facilities	+	V	Human settlement environment	Industry friendliness	+	I/II/III
Financial capital position	Credit opportunity	+	I/II/III		Greening degree of village	+	I/II
	Annual household income	+	I/II/III		Sewage and waste treatment	+	I/III
	Diversity of income	+	V		Environmental sanitation remediation	+	I/III
	Household deposits	+	V		Convenience of transportation facilities	+	I/II/III
Tourism capital status	Tourism resources	+	I/IV [50]		Village road hardening rate	+	IV [53]
	Tourism service capability	+	I/IV [50]		safe drinking water ratio	+	I/II/III
	Brand awareness	+	IV [50]		Penetration rate of sanitary toilets	+	I/II/III
	Tourism business housing	+	IV		Completeness of public facilities	+	I/II/III
	Fixed assets for tourism operation	+	IV [50]		Communication penetration rate	+	I/II/III
					Convenience of financial services	+	I/II/III
					Satisfaction with housing situation	+	IV [53]
**Social subsystem**							
Level of social construction	Kindergarten enrollment rate	+	I/II/III	Social equity	Illiteracy rate	−	I/II/V
	Formal education level	+	I/II/IV		Years of education per capita	+	I/II/III
	Training participation rate	+	I/II/III		Participation in job training	+	I/II/III
	Green education	+	I/II/IV [54]		Mastery of skills	+	I/II/III
	Health care services	+	I/II/III		Integration of human connections	+	V
	Women in Management	+	I/II/III	Status of social capital	Social welfare	+	I/II/V
	Neighborhood	+	IV [54]		Degree of social connectedness	+	V
Level of social governance	Price transparency	+	I/II		Skills Training Opportunities	+	I/II/V
	Policy tolerance	+	I/II		Degree of social support	+	I/II/V
	Management fairness	+	I/II/III		Degree of location advantage	+	I/II/V
	Government integrity	+	I/II	Human capital status	Number of farmers	+	I/II/III/V
	Villagers’ participation	+	IV [54]		Family size	+	V
	Satisfaction with service	+	I/II/III		Family labor Capacity	+	I/II/V
	The judicial relief	+	I/II/III		Health of family members	+	I/II/V
	Public order stability index	+	I/II/III		Educational level of family members	+	I/II/V
	Satisfaction with life	+	I/II/III		Subsistence non-agricultural level	+	V
	Residents’ well-being	+	IV [53]	Status of Cultural Capital	Cultural awareness level	+	IV [55]
	Relative deprivation	−	III		Cultural use level	+	IV [55]
	Population settlement rate	+	I/II/III		Cultural enjoyment level	+	IV [55]
	Indigenous permanent residence rate	+	I/II/III				

Note: I: IAEG-SDGs; II: Country programmer; III: Progress report; IV: Literature; V: SLA.

**Table 2:** Expert Judgment Basis and Assignment.

Judgment Basis	Degree of Influence on Expert Judgment		
	High	Middle	Low
The theoretical analysis	0.40	0.30	0.20
Practical experience	0.40	0.30	0.20
Peers to understand	0.10	0.10	0.10
Intuition	0.10	0.10	0.10

**Table 3:** Evaluation system for sustainable livelihood of farmers in ecological resettlement area in the upper reaches of the Yellow River.

First-Level Indicator	Serial Number	Secondary Indicators	Weight	Source	First-Level Indicator	Serial Number	Secondary Indicators	Weight	Source
Economic subsystem					Environment subsystem				
Economic benefit (0.0405)	A1	Agricultural income share	0.0115	Government	Natural capital status (0.4242)	G1	Cultivated land resources	0.0928	Government
	A2	Engel coefficient	0.0136	Government		G2	Forest Resources	0.0892	Government
	A3	Proportion of expenditure on culture, education and entertainment services	0.0079	Sampling		G3	Grassland resources	0.1205	Government
	A4	Private car ownership	0.0075	Sampling		G4	Water resources	0.1217	Government
Resistance to risk (0.0681)	B1	Household debt ratio	0.0081	Sampling	Environmental bearing condition (0.4063)	H1	The population density	0.0126	Government
	B2	Labor employment rate	0.0088	Government		H2	Ecological carrying capacity	0.0213	Government
	B3	Labor insurance coverage	0.0085	Government		H3	Per capita cultivated land area	0.0223	Government
	B4	Total grain production	0.0088	Government		H4	Per capita water resources	0.0171	Government
	B5	GDP per capita	0.0090	Government		H5	Environmental protection investment intensity	0.0167	Government
	B6	Economic growth rate	0.0078	Government		H6	Ecological footprint	0.0213	Government
	B7	Urban-rural income gap	0.0094	Government	Climate change and impacts (0.0462)	I1	Impact of water resources shortage	0.0081	Interview
	B8	Community gap between rich and poor	0.0077	Government		I2	Impact of desertification	0.0127	Interview
Financial capital position (0.2316)	C1	Credit opportunity	0.0583	Government		I3	Incidence of meteorological disasters	0.0127	Government
	C2	Annual household income	0.0573	PRA		I4	Disaster prevention capacity	0.0127	Interview
	C3	Diversity of income	0.0568	PRA	Environment adaptation level (0.1233)	J1	Satisfaction rate of agricultural land quality	0.0218	PRA
	C4	Household deposits	0.0592	PRA		J2	Environmental satisfaction rate	0.0347	PRA
Physical capital status (0.4689)	D1	Fixed household assets	0.1320	PRA		J3	Traffic satisfaction	0.0347	PRA
	D2	Area of homestead	0.1135	PRA		J4	Satisfaction with community security	0.0320	PRA
	D3	Livestock breeding	0.1024	PRA	Human settlement environment (0.1881)	K1	Industry friendliness	0.0192	PRA
	D4	Family living facilities	0.1209	PRA		K2	Environmental sanitation remediation	0.0247	PRA
Tourism capital status (0.0552)	E1	Tourism resources	0.0150	PRA		K3	Convenience of transportation facilities	0.0212	PRA
	E2	Fixed assets for tourism operation	0.0240	Interview		K4	Village road hardening rate	0.0265	Government
	E3	Tourism service capability	0.0162	PRA		K5	safe drinking water ratio	0.0159	PRA
Development condition (0.1380)	F1	Characteristic industry development	0.0483	Interview		K6	Completeness of public facilities	0.0275	PRA
	F2	Industrial diversification	0.0390	Sampling		K7	Convenience of financial services	0.0260	PRA
	F3	Livelihood diversity index	0.0507	Interview		K8	Satisfaction with housing situation	0.0270	PRA
Social subsystem									
Level of social governance (0.1500)	L1	Policy transparency	0.0127	Sampling	Social equity (0774)	O1	Illiteracy rate	0.0147	Government
	L2	Policy tolerance	0.0105	Sampling		O2	Years of education per capita	0.0141	Government
	L3	Management fairness	0.0112	Sampling		O3	Participation in job training	0.0150	PRA
	L4	Government integrity	0.0122	Sampling		O4	Mastery of skills	0.0143	PRA
	L5	Villagers’ participation	0.0107	Sampling		O5	Integration of human connections	0.0193	Interview
	L6	Satisfaction with service	0.0107	Sampling	Status of social capital (0.3645)	P1	Social welfare	0.0735	Government
	L7	The judicial relief	0.0108	Government		P2	Degree of social connectedness	0.0626	Interview
	L8	Public order stability index	0.0108	Sampling		P3	Skills Training Opportunities	0.0757	PRA
	L9	Satisfaction with life	0.0127	Sampling		P4	Degree of social support	0.0793	Government
	L10	Residents’ well-being	0.0127	Sampling		P5	Degree of location advantage	0.0735	Interview
	L11	Relative deprivation	0.0126	Sampling	Human capital status (0.3711)	Q1	Number of farmers	0.0658	Government
	L12	Population settlement rate	0.0116	Interview		Q2	Family size	0.0683	PRA
	L13	Indigenous permanent residence rate	0.0109	Interview		Q3	Family labor Capacity	0.0658	PRA
Level of social construction (1041)	M1	Formal education level	0.0521	Sampling		Q4	Health of family members	0.0434	PRA
	M2	Health care services	0.0521	Interview		Q5	Educational level of family members	0.0594	PRA
Status of Cultural Capital (0.0375)	N1	Cultural awareness level	0.0181	Interview		Q6	Subsistence non-agricultural level	0.0683	Sampling
	N2	Cultural use level	0.0194	Interview					

**Table 4:** Classification criteria for comprehensive evaluation index, coordination level and coordination development degree.

Section	[0, 0.1)	[0.1, 0.2)	[0.2, 0.3)	[0.3, 0.4)	[0.4, 0.5)	[0.5, 0.6)	[0.6, 0.7)	[0.7, 0.8)	[0.8, 0.9)	[0.9, 1]
W and K	Very bad	Bad	Very worse	Worse	Very poor	Poor	Qualified	Good	Excellent	Superexcellent
R	Extreme maladjustment	Severe maladjustment	Moderate maladjustment	Mild maladjustment	On the verge of maladjustment	Grudging coordination	Primary coordination	Intermediate coordination	Good coordination	High quality coordination
J	Fading type	Fading type	Fading type	Fading type	Fading type	Type of development	Type of development	Type of development	Type of development	Type of development
Colour										
Major categories	Type of maladjustment recession				Type of transitional development			Types of coordinated development		

**Table 5:** Basic information of empirical objects of ecological migration area in Liujiaxia Reservoir Area.

The Administrative Area	Leading Industry	The Administrative Area	Leading Industry
Taiji Town	Tourism industry	Sanyuan Town	Planting/breeding
Liujiaxia Town	Tourism industry	Santiao Xian Town	Planting/breeding
Yanguoxia Town	Tourism industry	Xinsi Town	Planting/breeding
Hongquan Town	Planting/breeding	Yangta Town	Planting/breeding

**Table 6:** The characteristics of interviewees.

Type	Household Size (Person/Household)	Number of Households (Person/Household)	Number of Migrant Workers (Person/Household)	Annual Household Income (Yuan)	Education Level of Labor Force (%)			
					Illiteracy	Primary School	Junior High School	High School or Above
Not a file card	4.42	3.45	1.63	38,845	34.76	23.65	21.83	19.76
File a card	4.14	3.23	1.54	41,265	42.55	31.60	16.35	9.5

**Table 7:** Comprehensive evaluation results and coordinated development types of ecological migration areas in Liujiaxia Reservoir Area.

Township	Liujiaxia	Yanguoxia	Taiji	Xihe	Xianyuan	Sanyuan	Yangta
K	0.8437	0.8302	0.8451	0.8219	0.7605	0.7509	0.7441
J	0.7493	0.7389	0.7544	0.7382	0.7451	0.7507	0.7119
Township	Pinggou	Hongquan	Wangtai	Xiaoling	Xinsi	Santiaoxian	Chuancheng
K	0.7458	0.7514	0.7509	0.7435	0.7585	0.7586	0.7472
J	0.7199	0.7359	0.7366	0.7281	0.7460	0.7395	0.7184

Note: The coordination level corresponding to the colors in the table refers to the grading standard in Table 4.

**Table 8:** Moran’s *I* Table of Sustainable Livelihood Capital of Farmers in Ecological Migration Area of Liujiaxia Reservoir Area.

Living System	Moran’s *I*	z	*p*
Economic	0.5684	2.8161	0.0049
Social	0.3166	1.6869	0.0916

## Data Availability

Data will be made available on request.
